# Fertiliser management effects on dissolved inorganic nitrogen in runoff from Australian sugarcane farms

**DOI:** 10.1007/s10661-017-6115-z

**Published:** 2017-07-21

**Authors:** Grant Fraser, Ken Rohde, Mark Silburn

**Affiliations:** 1 Department of Science, Information Technology and Innovation, Queensland, Australia; 2 Department of Natural Resources and Mines, Queensland, Australia; 3 National Centre for Engineering in Agriculture, University of Southern Queensland, Queensland, Australia

**Keywords:** Nitrogen fertiliser application rates, Climate forecasting, Management, Water quality, Sugarcane, Great Barrier Reef

## Abstract

Dissolved inorganic nitrogen (DIN) movement from Australian sugarcane farms is believed to be a major cause of crown-of-thorns starfish outbreaks which have reduced the Great Barrier Reef coral cover by ~21% (1985–2012). We develop a daily model of DIN concentration in runoff based on >200 field monitored runoff events. Runoff DIN concentrations were related to nitrogen fertiliser application rates and decreased after application with time and cumulative rainfall. Runoff after liquid fertiliser applications had higher initial DIN concentrations, though these concentrations diminished more rapidly in comparison to granular fertiliser applications. The model was validated using an independent field dataset and provided reasonable estimates of runoff DIN concentrations based on a number of modelling efficiency score results. The runoff DIN concentration model was combined with a water balance cropping model to investigate temporal aspects of sugarcane fertiliser management. Nitrogen fertiliser application in December (start of wet season) had the highest risk of DIN movement, and this was further exacerbated in years with a climate forecast for ‘wet’ seasonal conditions. The potential utility of a climate forecasting system to predict forthcoming wet months and hence DIN loss risk is demonstrated. Earlier fertiliser application or reducing fertiliser application rates in seasons with a wet climate forecast may markedly reduce runoff DIN loads; however, it is recommended that these findings be tested at a broader scale.

## Introduction

### Dissolved inorganic nitrogen and the Great Barrier Reef

**Fig. 1:**
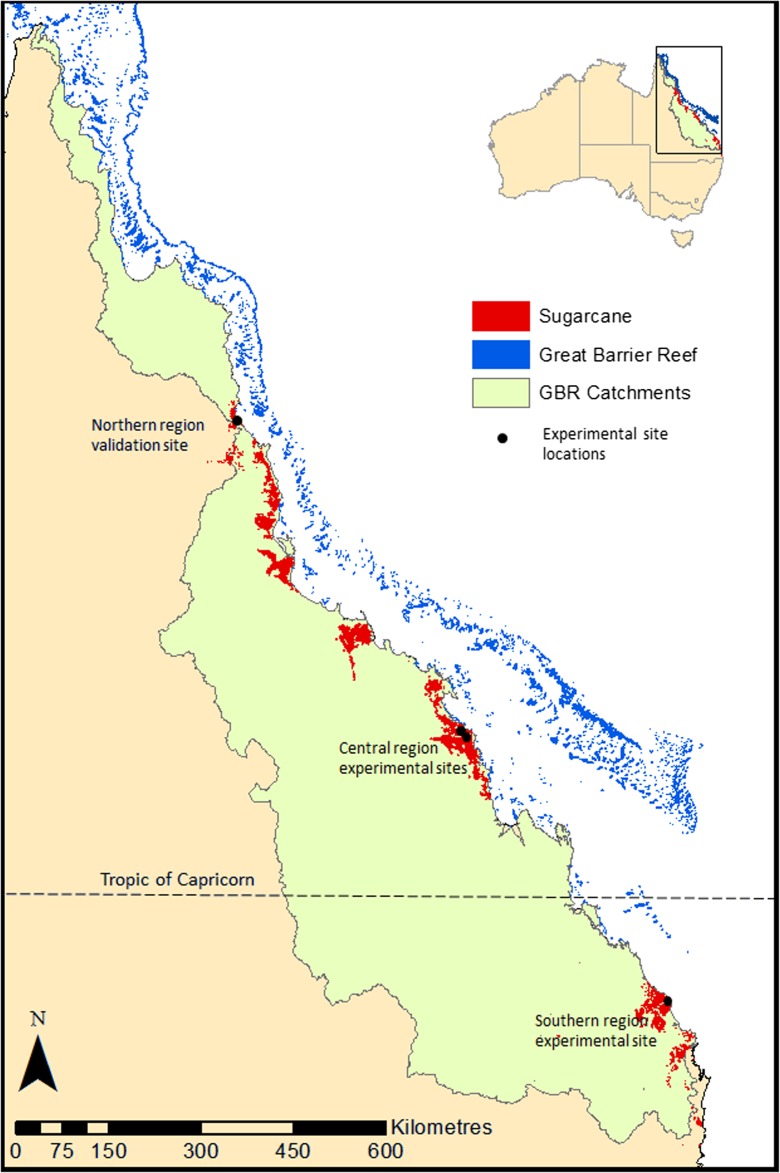
Australian sugarcane-growing regions that drain into the Great Barrier Reef. Locations of experimental sites are shown

Movement of dissolved inorganic nitrogen (DIN) from fertilised agricultural lands has been identified as a major water quality issue internationally (e.g. Howarth et al. 2002; Schindler et al. 2006; Swaney et al. 2012) and within Australia (e.g. Mitchell et al. 2009; Kroon et al. 2012; Thorburn et al. 2013). Elevated levels of DIN in runoff to the Great Barrier Reef (GBR) are believed to be responsible for large increases (10×) in phytoplankton which leads to an increase in larval stage survival of crown-of-thorns starfish (*Acanthaster planci*) and the potential for crown-of-thorns outbreaks (Brodie et al. 2005). Crown-of-thorns is estimated to have caused a ~21% reduction in GBR coral cover between the years 1985–2012 (De’ath et al. 2012). Sugarcane production occurs in coastal catchments adjoining the GBR (Fig. 1), and it is estimated that runoff DIN loads to the GBR have increased by threefold since pre-European times (Kroon et al. 2012). Catchments dominated by sugarcane production were found to have the highest median DIN runoff concentrations when compared to a range of other agricultural and non-agricultural land uses (Bartley et al. 2012). Waters et al. (2014) estimated that sugarcane contributes 68% of the anthropogenic DIN load to the GBR. To protect the GBR, the Australian government has implemented a Reef Water Quality Protection Plan program which aims to reduce anthropogenic DIN loads to the GBR by 50% through improving land management practices (Reef Water Quality Protection Plan Secretariat 2013). As sugarcane has the highest concentrations of DIN in runoff and is in close proximity to the GBR, sugarcane farm management of nitrogen fertiliser has been a major focus of the government initiative. To support the program, there have been a number of experiments monitoring DIN runoff loads from sugarcane farms.

### Australian sugarcane farming and fertiliser management practices

In Australia, sugarcane is grown across a range of environments from wet tropics (e.g. northern region, Innisfail—3500 mm), dry tropics (e.g. southern section of northern region, Ayr 1000 mm) through to sub-tropics (e.g. southern region, Childers 880 mm). All regions have high inter-annual rainfall variability (average coefficient of variation of 34%) which is in part, influenced by the El Niño-Southern Oscillation (Allan 1985) and the Pacific Decadal Oscillation (Power et al. 1999). Years with high rainfall can lead to large quantities of runoff and should therefore be the primary focus for management of constituent load generation. Additionally, sugarcane yields can halve under wet conditions (e.g. Schroeder et al. 2010), due to either waterlogging (Rudd and Chardon 1977) or as a result of a reduction in solar radiation (e.g. Inman-Bamber 1995). Hence, sugarcane nitrogen requirements are also potentially reduced in particularly wet years.

Australian sugarcane cropping practices follow a 5- to 6-year cycle. The crop is harvested on a ~12- to 13-month time period. After each harvest, the crop re-establishes from the same rootstock (ratooning) until the third or fourth ratoon whereupon the rootstock is ploughed out. After the final ratoon stage, the land is either left as a bare fallow or a legume such as soybeans or cowpeas is sown. Nitrogen (N) fertiliser is typically applied twice in the plant stage—an initial small amount at planting (e.g. 30 kg N/ha) followed by a top-up ~3 months after planting (e.g. 130 kg N/ha). Fertiliser is often applied only once during each ratoon crop with rates being in the range of 130–200 kg N/ha, depending on the productivity of the region. Fertiliser application generally occurs in the 5-month period from late winter (August) through to early summer (December). These time frames are associated with the cane harvesting and planting schedule which occurs during the same time period. There are a number of factors that can affect the timing of fertiliser applications. These include date of harvesting of the previous crop, crop height for machinery access and weather conditions which can affect tractor access.

### Australian sugarcane measured runoff DIN loads

Field-measured DIN in runoff provides an opportunity to identify the major impacts of sugarcane farming management practices on DIN loads. Table 1 shows monitored DIN runoff loads on an annual basis and associated experimental details from eight on-farm monitoring experiments. Catchments were in the range of 1500–4300 m^2^, except for at Ayr where catchments were up to 11.6 ha. DIN loads in runoff vary from being 0.1–11% of applied nitrogen fertiliser, with a median loss of 1.3% of applied nitrogen fertiliser (hence having little to no agronomic impact). Understanding the causes of the variability in DIN loads may inform better fertiliser management strategies to minimise DIN losses in runoff. The fate of nitrogen fertiliser in cane farming systems is complex with 11 pathways described by Bristow et al. (1998). However, for most of the sugarcane farm experiments in Table 1 (and for the experiments analysed subsequently), only the runoff DIN pathway has been measured on a daily basis. Processes such as fertiliser transformations, soil N immobilisation/mineralisation, N leaching, denitrification and plant N uptake have either not been measured or have been measured infrequently. Hence, the development of daily DIN runoff loss model from these datasets cannot aim to represent the variety of soil nitrogen processes known to occur. Nevertheless, these experimental studies do provide information with relation to the range of DIN concentrations in runoff with respect to fertiliser application rates, fertiliser form, timing and rainfall since application. In this study, we develop a daily time-step empirical model of DIN concentrations in runoff that can be easily incorporated into paddock-scale hydrological models and which represent the effects of the main management practices available to sugarcane growers, i.e. N rate, N form and timing of application. This runoff DIN concentration model is incorporated into an agricultural water balance and crop growth model to explore the impacts of management using a long-term climate sequence.

**Table 1:** Annual DIN runoff loads measured from Australian sugarcane field experimental plots

Data number	Location	Crop stage	Year	Apply method	Nitrogen input (kg/ha)	DIN load (kg/ha)	Study reference
1	Mossman (NWT)	Third ratoon	2003–2004	SUR	186	2.0	Webster et al. (2012)
2	Mossman (NWT)	Third ratoon	2003–2004	SUR	102	0.6	Webster et al. (2012)
3	Mossman (NWT)	Fourth ratoon	2004–2005	SUR	179	2.3	Webster et al. (2012)
4	Mossman (NWT)	Fourth ratoon	2004–2005	SS	86	1.1	Webster et al. (2012)
5	Mossman (NWT)	Fifth ratoon	2005–2006	SUR	175	1.8	Webster et al. (2012)
6	Mossman (NWT)	Fifth ratoon	2005–2006	SUR	96	0.6	Webster et al. (2012)
7	Tully (NWT)	Fallow	2009–2010		0	0.1	Armour et al. (2013)
8	Tully (NWT)	Fallow	2009–2010		0	<0.1	Armour et al. (2013)
9	Tully (NWT)	Plant	2010–2011	SS	15	0.2	Armour et al. (2013)
10	Tully (NWT)	Plant	2010–2011	SS	95	0.8	Armour et al. (2013)
11	Tully (NWT)	First ratoon	2011–2012	SS	129	0.6	Armour et al. (2013)
12	Tully (NWT)	First ratoon	2011–2012	SS	131	2.0	Armour et al. (2013)
13	Tully (NWT)	Second ratoon	2012–2013	SS	130	4.1	Armour et al. (2013)
14	Tully (NWT)	Second ratoon	2012–2013	SS	138	5.4	Armour et al. (2013)
15	Ingham (NWT)	Plant	2010–2011	SS	96	1.9	Armour et al. (2012)
16	Ingham (NWT)	Plant	2010–2011	S	96	0.7	Armour et al. (2012)
17	Ingham (NWT)	Plant	2013–2014	SS	120	0.6	Royle (2016)
18	Ingham (NWT)	Plant	2013–2014	SS	120	1.4	Royle (2016)
19	Ingham (NWT)	First ratoon	2014–2015	SS	160	<0.1	Royle (2016)
20	Ingham (NWT)	First ratoon	2014–2015	SS	160	0.1	Royle (2016)
21	Ayr (NDT)	First ratoon	2005–2006	SS	219	~1^a^	Thorburn et al. (2011a)
22	Ayr (NDT)	First ratoon	2004–2005	SS	234	~5^a^	Thorburn et al. (2011a)
23	Ayr (NDT)	Third ratoon	2011–2012	SS	170	6.3^c^	Davis et al. (2012)
24	Ayr (NDT)	Second ratoon	2011–2012	SS	170	0.1	Davis et al. (2012)
25	Mackay (CR)	Plant	2009–2010	SS	133	14.6^b^	Rohde et al. (2013a)
26	Mackay (CR)	Plant	2009–2010	SS	38	7.6^b^	Rohde et al. (2013a)
27	Mackay (CR)	First ratoon	2010–2011	S	200	5.8	Rohde et al. (2013a)
28	Mackay (CR)	First ratoon	2010–2011	S	136	6.3	Rohde et al. (2013a)
29	Mackay (CR)	Second ratoon	2011–2012	S	200	1.5	Rohde et al. (2013a)
30	Mackay (CR)	Second ratoon	2011–2012	S	139	1.5	Rohde et al. (2013a)
31	Mackay (CR)	Third ratoon	2012–2013	S	197	4.1	Rohde et al. (2013b)
32	Mackay (CR)	Third ratoon	2012–2013	S	135	1.6	Rohde et al. (2013b)
33	Mackay (CR)	Third ratoon	2012–2013	S	197	0.9	Rohde et al. (2013b)
34	Mackay (CR)	Third ratoon	2012–2013	S	135	1.4	Rohde et al. (2013b)
35	Mackay (CR)	Fourth ratoon	2013–2014	S	200	0.7	Chataway et al. (2014)
36	Mackay (CR)	Fourth ratoon	2013–2014	S	135	0.9	Chataway et al. (2014)
37	Mackay (CR)	Fourth ratoon	2013–2014	S	200	1.0	Chataway et al. (2014)
38	Mackay (CR)	Fourth ratoon	2013–2014	S	135	0.3	Chataway et al. (2014)
39	Bundaberg (SR)	Plant	2011–2012	SS	146	2.2	Nachimuthu et al. (2012)
40	Bundaberg (SR)	Plant	2011–2012	SS	146	0.8	Nachimuthu et al. (2012)
41	Bundaberg (SR)	Plant	2011–2012	SS	146	0.2	Nachimuthu et al. (2012)
42	Bundaberg (SR)	Plant	2011–2012	SS	146	0.7	Nachimuthu et al. (2012)
Average (excluding years 7, 8)					144	2.3	
Median (excluding years 7, 8)					135	1.3	

*NWT* north region wet tropics, *NDT* north region dry tropics, *CR* central region, *SR* southern region, *S* surface, *SUR* surface under residue, *SS* sub-surface

^a^Nitrate only

^b^Soybean fallow estimated to contribute 300 kg N/ha

^c^Total for November–February

## Methods

### Experimental site characteristics

DIN concentrations were measured in surface runoff from three sugarcane experimental trials (Fig. 1) at a paddock scale with catchment areas ranging from 809 to 1569 m^2^. Two sites (Victoria Plains and Marian) were located in the central sugarcane growing region, and a third site (Wallum Creek) was located in the southern sugarcane growing region. Annual load results for the Victoria Plains site are reported in Table 1, data numbers 25–38. Load measurements were not calculated for the Marian site due to problems with estimating large runoff event volumes. Each site had between two and five runoff plots with flumes and refrigerated pumping samplers. Additionally, each site was located on a different soil type—a sandy–loam soil with a seasonal perched water table (Hydrosol), Brown Chromosol and a self-mulching medium clay (Vertosol) (Table 2). There was a large difference in rainfall measured during the trial period between the two sites in the central region (annual average ~2600 mm) and the southern site (annual average—961 mm) (Table 2). The sugarcane harvest residue management also varied, with the cane being burnt at the southern site and retention of green cane residue (locally called green trash blanketing) at the central region sites. Further detailed descriptions of the site characteristics, experimental details, farm management practices and hydrology can be found in (Simpson 2001; Rohde et al. 2013a, b). Fertiliser application dates, fertiliser nitrogen input rates and the form of nitrogen fertiliser were collected for each site (Table 3).

**Table 2:** Characteristics of the three sites and experimental conditions

Site	Soil classification^a^	Years of measurement	Average annual rainfall during trial (mm)	Plot catchment area (m^2^)	Trash management	Number of fertiliser treatments	Number of replicates
Wallum Creek (SR)	Redoxic Hydrosol	1998–2000	961	809	Burnt	1	2
Victoria Plains (CR)	Black–Dark Grey Vertosol	2009–2012	2582	1415	Green cane trash blanket	2	1
Marian (CR)	Brown Chromosol	2009–2012	2626	1569	Green cane trash blanket	5	1

*SR* southern region, *CR* central region

^a^Australian soil classification (Isbell 1996)

**Table 3:** Type of fertiliser, number of applications, nitrogen application rate and associated runoff events with DIN measurements

Year combination	Site	Year	Application 1	Application 2	Total nitrogen Input (kg/ha)	Number of runoff events with measured DIN
			Fertiliser, form, rate (kg/ha)	Fertiliser, form, rate (kg/ha)		
1	Wallum Creek Plot 1	1998–1999	Urea–ammonium sulphate, G, 167		167	5
2	Wallum Creek Plot 2	1998–1999	Urea–ammonium sulphate, G, 167		167	4
3	Wallum Creek Plot 1	1999–2000	Urea–ammonium sulphate, G, 178		178	7
4	Wallum Creek Plot 2	1999–2000	Urea–ammonium sulphate, G, 178		178	3
5	Victoria Plains Treatment 1	2009–2010	Soybean crop residue, 300	Diammonium phosphate, G, 38	433	12
6	Victoria Plains Treatment 2	2009–2010	Soybean crop residue, 300	Diammonium phosphate, G, 38	338	10
7	Victoria Plains Treatment 1	2010–2011	Urea–biodunder, L, 200		200	23
8	Victoria Plains Treatment 2	2010–2011	Urea–biodunder, L, 137		137	18
9	Victoria Plains Treatment 1	2011–2012	Urea–biodunder, L, 201		201	11
10	Victoria Plains Treatment 2	2011–2012	Urea–biodunder, L, 139		139	6
11	Marian Treatment 1	2009–2010	Diammonium phosphate, G, 45	Urea–ammonium sulphate, G, 146	191	4
12	Marian Treatment 2	2009–2010	Diammonium phosphate, G, 45	Urea–ammonium sulphate, G, 146	191	10
13	Marian Treatment 3	2009–2010	Diammonium phosphate, G, 45	Urea–ammonium sulphate, G, 127	172	8
14	Marian Treatment 4	2009–2010	Diammonium phosphate, G, 45	Urea–ammonium sulphate, G, 27	97	4
15	Marian Treatment 5	2009–2010	Diammonium phosphate, G, 45	Urea–ammonium sulphate, G, 119	164	7
16	Marian Treatment 1	2010–2011	Urea–biodunder, L, 197	Ammonium sulphate, G, 61	258	8
17	Marian Treatment 2	2010–2011	Urea–biodunder, L, 197	Ammonium sulphate, G, 61	258	17
18	Marian Treatment 3	2010–2011	Urea–biodunder, L, 159	Ammonium sulphate, G, 61	220	18
19	Marian Treatment 4	2010–2011	Urea–biodunder, L, 119	Ammonium sulphate, G, 61	180	15
20	Marian Treatment 5	2010–2011	Urea–biodunder, L, 159	Ammonium sulphate, G, 61	220	17
21	Marian Treatment 1	2011–2012	Urea–biodunder, L, 197		197	6
22	Marian Treatment 2	2011–2012	Urea–biodunder, L, 197		197	9
23	Marian Treatment 3	2011–2012	Urea–biodunder, L,159		159	6
24	Marian Treatment 4	2011–2012	Urea–biodunder, L, 53		53	6
25	Marian Treatment 5	2011–2012	Urea–biodunder, L, 159		159	3
Total						237

A third application occurred in year 5—urea, granular, 95 and year 14—ammonium sulphate, granular, 25

*G* granular, *L* liquid

### Fertiliser management practices during the experiments

The form of nitrogen fertiliser (granular or liquid) and application rates varied between sites, as well as between years for a given site (Table 3). All fertiliser applications were applied to the surface. There were multiple fertiliser applications per season in some years at the central region farms. In years 16–20, a mix of liquid and granular fertiliser was used; however, the majority of the nitrogen (average 73%) was applied in liquid form; for the purposes of categorising each year as being either granular or liquid form, we considered these years to be liquid form. In the first season (2009–2010) at Victoria Plains, plant cane was established and was preceded by a fallow soybean crop that was incorporated into the soil (to a depth of 10 cm approximately 6 weeks after being sprayed out with glyphosate herbicide). Although nitrogen content of the soybean crop was not measured at the time of incorporation, the contribution of nitrogen was estimated to be 300 kg/ha (Schroeder et al. 2005). This contribution of nitrogen to the system may provide a large proportion of the N requirements for the forthcoming cane crop (e.g. Park et al. 2010). The nitrogen contribution from the soybean crop will now be considered on the same basis as nitrogen contributed from fertiliser applications. One of the commonly applied fertilisers at the central region sites was a surface applied, low concentrate liquid nitrogen and biodunder mix. Biodunder is an organic waste by-product from the cane mill sugar refining process and is usually re-applied to cane fields as a fertiliser. The biodunder component contributed 0.5% nitrogen to these mixes and the remaining nitrogen came from the addition of liquefied urea.

The number of runoff events with DIN concentration measurements varied greatly across sites and between years, with the 2010–2011 season at the central region sites resulting in the greatest number of runoff events with DIN measurements.

To validate the proposed granular fertiliser model, an independent dataset from a sugarcane site located in the northern cane growing region was used (Webster et al. 2012). At this site, runoff DIN concentrations were measured over three seasons for two nitrogen fertiliser treatments. One fertiliser treatment was based on local standard industry practices (N farm—Webster et al. 2012), and the nitrogen fertiliser input for the three seasons was 186, 179 and 175 kg/ha each applied as a single surface application of urea prills. The second nitrogen fertiliser treatment was based on ‘replacement’ of nitrogen (N repl—Webster et al. 2012) lost as a result of harvest of the previous year’s crop (Thorburn et al. 2011b). In this treatment, nitrogen fertiliser input for the three seasons was 102, 86 and 96 kg/ha which was applied as a single surface application of urea prills in years 2004, 2006 and sub-surface (0.1 m) in 2005. The average seasonal rainfall from fertiliser application to early June, the following year was 2393 mm (similar to two of the calibration sites) and average seasonal runoff was 548 mm. Over the three seasons, 75 runoff events were recorded.

### Calibration and application of the HowLeaky model for Victoria Plains

The Victoria Plains sugarcane runoff trial was modelled using the cropping systems model HowLeaky (Freebairn et al. 2003; Thornton et al. 2007; Robinson et al. 2010). HowLeaky simulates the one-dimensional components of the water balance on a daily basis, has a simple crop growth model and was primarily designed to model management impacts on runoff and constituent movement (sediment, pesticides and phosphorus). The objective of the calibration procedure was to attain a representation of daily runoff processes at the site taking into account measured soil characteristics and crop water use. The calibrated model was subsequently used to simulate runoff for the site using a long term climate data sequence.

Field measurements taken during the 3 years of field trial included: daily rainfall; irrigation; soil water content; daily surface runoff; and end of season crop biomass yields. A HowLeaky soil parameter file developed to describe a moderately deep, slowly permeable, Vertosol for the Australian Governments paddock modelling program provided the starting basis for modelling this site (Shaw et al. 2013). The soil water measurements taken during the trial were used to set the upper and lower soil water limits in the model. Additionally, the soil water drainage rate from the bottom soil layer at 2 m depth was reduced from 5 to 2 mm/day to represent the presence of a water table which was observed during particularly wet rainfall periods (Table 4). A major factor determining daily runoff rates in HowLeaky is the runoff curve number, which was left at the default setting for this soil type of 78. This resulted in a coefficient of determination of 0.88 between daily observed runoff and daily modelled runoff (Table 4). The HowLeaky model can be parameterised to represent daily sugarcane crop growth by characterising leaf area development, biomass accumulation and root growth. The key parameter—crop radiation use efficiency—was set to 2.5 g/m^2^/MJ which was between the 2–3 g/m^2^/MJ recommended in the HowLeaky. Modelled and observed end of season dry matter yields are shown in Table 4. Planting and harvest dates were set as undertaken in the trial. The only model adjustment made for the subsequent simulation study was to apply 50 mm of irrigation when the soil water deficit was >75 mm with there being at least 15 days between irrigations. This rule was made in the simulation study to represent a farmer irrigating the crop. The simulation study was conducted using the climate record from Pleystowe sugar mill located near the central region sites for the years 1901–2012.

**Table 4:** Observed and calibrated HowLeaky model runoff, waterlogging and cane yields on a seasonal basis

Season	2009–2010	2010–2011	2011–2012
Observed runoff (mm)	815	2020	950
Predicted runoff (mm)	978	2069	1202
Observed days waterlogged	58	177	109
Predicted days waterlogged	78	164	77
Observed yield (dry matter kg/ha)	41,824	21,265	39,882
Predicted yield (dry matter kg/ha)	40,418	23,255	38,119

### Variability in sugarcane yield and solar radiation

To investigate the effect of solar radiation on cane yields, we accumulated the September–June daily satellite derived solar radiation (Bureau of Meteorology 2009) for the four major cane growing regions in Australia. Daily satellite-derived solar radiation for each region was based on regionally averaging satellite derived daily solar radiation (Bureau of Meteorology 2009) from 0.05° grid cells, for cells with greater than 50% sugarcane land use. Sugarcane mill yield data presented in Schroeder et al. (2010) were aggregated for the four regions.

### Application of a climate forecast system to understand DIN runoff risk

**Fig. 2:**
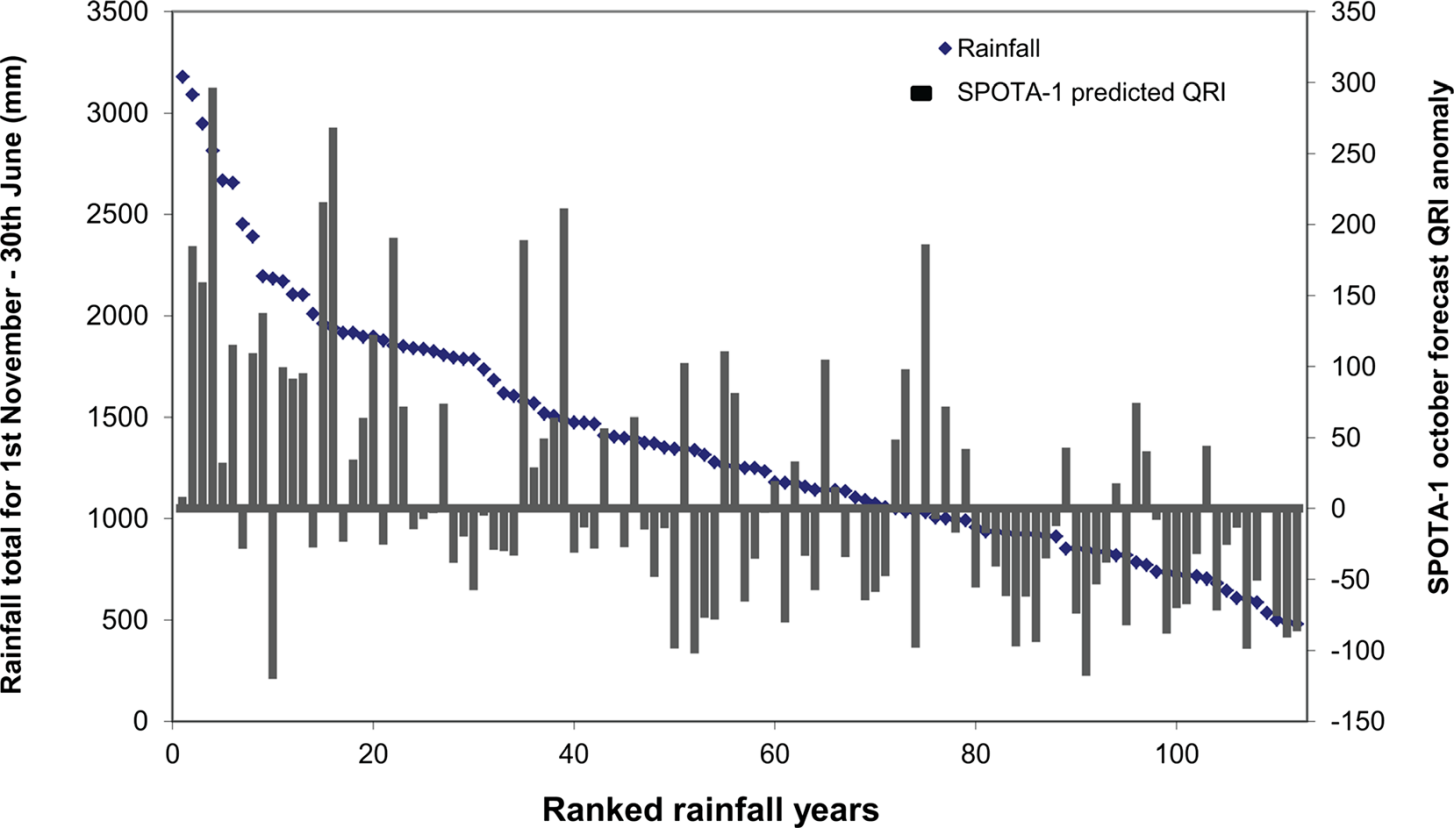
Accumulated November–June rainfall from Pleystowe station near Victoria Plains and the October SPOTA-1 forecast Queensland Rainfall Index anomaly

The SPOTA-1 (Seasonal Pacific Ocean Temperature Analysis - version 1) climate forecast system Day et al. 2001 was developed based on the relationships between a Queensland Rainfall Index (QRI) and two sea surface temperature indices for the period 1900–1996. The two sea surface indices are as follows: (1) Norfolk–Hawaii Index (a north–south sea surface temperature anomaly) and (2) South West Pacific Index (a south–west Pacific sea surface temperature anomaly). Independent operational forecasts have been provided online since April 2000 (http://www.longpaddock.qld.gov.au/about-spota1/about.html). SPOTA-1 forecasts are provided on a monthly basis from June through to November each year for the forthcoming seasonal rainfall for December to March. These climate forecasts align to when farmers need to make decisions regarding fertilising their sugarcane crop. Figure 2 shows the relationship between the SPOTA-1 forecast QRI anomaly and the forthcoming seasonal rainfall for the climate station used in the simulation study. While the SPOTA-1 forecast QRI anomaly was not always aligned well with the November–June observed rainfall, there is a relationship between rainfall and the forecast QRI anomaly (*r* ^2^ = 0.25, *p* < 0.05). In the simulation study, the October SPOTA-1 forecast QRI anomaly was classified as being in one of three categories: ‘wet’, ‘average’ and ‘dry’ forecast rainfall conditions. This system of classification was achieved by ranking the forecast QRI anomaly for the years 1900–2013, the top third of the rankings—i.e. those forecasting a wet summer—were assigned to the wet group. Simulated seasonal runoff DIN loads from HowLeaky were also accumulated for the same tercile groups. The simulation study examined the impact of granular and liquid fertiliser applied at close to conventional rates of 170 kg N/ha and at 85 kg N/ha (i.e. rates similar to the nitrogen replacement system developed by Thorburn et al. 2011b). The date of fertiliser application was the same for both fertiliser application rates.

## Results and interpretation

### Development of a model for daily DIN runoff concentration from monitored data

**Fig. 3:**
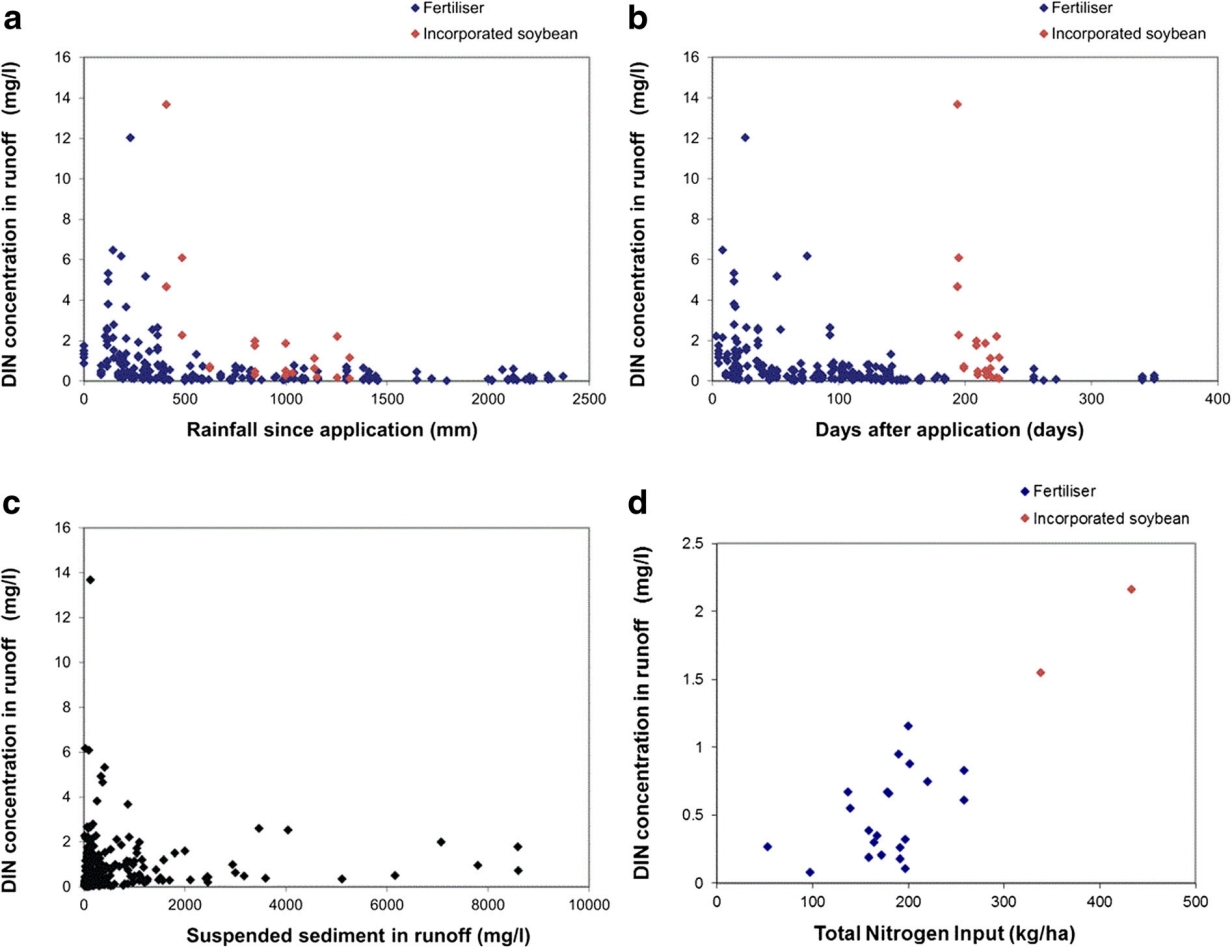
DIN concentration measured in runoff water relative to **a** rainfall since application; **b** days after application; **c** runoff suspended sediment concentrations; and **d** total nitrogen input

Factors investigated with respect to DIN concentrations in surface runoff were as follows: (a) rainfall since application (including irrigation events); (b) time after application; (c) sediment concentration in runoff; and (d) fertiliser N application rate (Fig. 3).

**Fig. 4:**
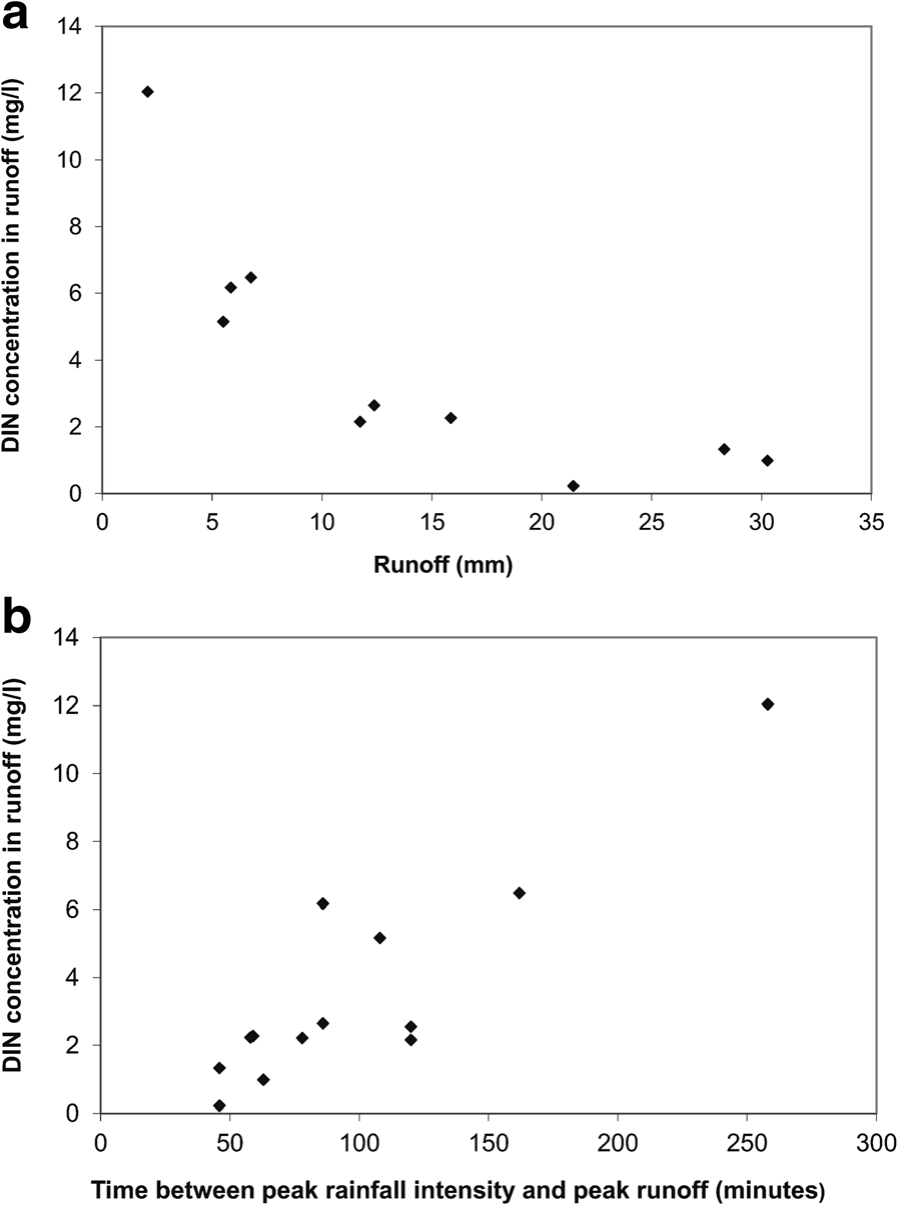
DIN concentrations for a number of runoff events in the time period between 50 and 100 days after fertiliser application. Intermittent increases in runoff DIN concentrations were associated with small runoff events (**a**) that also had an extended time between the peak rainfall rate and the peak runoff rate (**b**)

Upon visual inspection, three of these factors—time after application, rainfall since application and N application rate—had an effect on DIN concentrations in runoff, while suspended sediment did not appear to have any consistent effect. The incorporated soybean crop appeared to have a delayed effect on DIN in runoff when compared to fertilisers (Fig. 3a, b). In the case of applied fertilisers, after ~40 days or 400 mm of rainfall, there were almost no concentrations above ~1 mg/l (Fig. 3a, b). However, DIN concentrations did not always decrease sequentially for each subsequent runoff event, with a number of events having runoff DIN concentrations up to 10 times higher than earlier runoff events with low DIN concentrations (e.g. events between 50 and 100 days after application in Fig. 3b). Close inspection of the hydrological conditions for these runoff events revealed that the high DIN concentrations tended to be associated with events that resulted in small runoff quantities of less than 10 mm (Fig. 4a). Another feature of these runoff events was that the time between the events’ peak rainfall intensity and the peak runoff was generally greater than 60 min (Fig. 4b) which suggests an extended residence time for runoff water being in contact with the soil surface. This may allow for more time for surface soil DIN to be assimilated into the runoff water.

As these unusually high DIN concentration events had low runoff volumes, they contributed only a small proportion of the total DIN lost in runoff over a full season. Therefore, these runoff DIN concentration measurements were not included in any further model development (7 measurements from the total 237 runoff DIN measurements were excluded).

### DIN model development

Given that there were multiple nitrogen fertiliser applications in 12 of the 25 seasons (Table 3), runoff DIN concentrations could potentially be influenced by one or more fertiliser application depending on when they occurred in the season. In order to develop a DIN model that can be easily implemented in daily water balance models, we aimed to represent the DIN available for runoff as a single pool (i.e. after the first application, subsequent applications can be added to the pool). Given this model form, the performance of the model was assessed on a seasonal basis rather than for each individual DIN concentration. The latter would give an undue weighting to seasons with a large number of runoff events. Hence, model coefficients were optimised based on the model performance over a seasonal basis. Further refinement of the model was done iteratively based on how these model coefficients varied from season to season.

As both time and rainfall since application may be correlated, two independent empirical models of DIN concentrations were developed based on these two components and a third empirical model was developed by combining both these components. Given the occurrence of multiple inputs of nitrogen for a number of seasons at the central region sites, a daily time-step model of DIN concentration in runoff was developed. The three models were—(1) time after application and application rate, (2) rainfall since application and application rate and (3) time after application and rainfall since application and application rate.

Rainfall has been reported to contain low levels of DIN; hence, we set a lower DIN concentration limit for the runoff models. There have been relatively few reported measurements of naturally occurring background levels of DIN in rainfall for coastal areas in Queensland. In the Townsville region, Probert (1976) measured ~2.4 kg DIN/ha/year. Hurditch and Charley (1982) reported a range in measurements from 21 kg DIN/ha/year close to the ocean to 6 kg DIN/ha/year some 2 km inland in southern Queensland. Taking into account the rainfall quantity at these locations suggests that at the lower end DIN concentrations in rainfall were between 0.02 and 0.045 mg/l. For all recorded runoff at the experimental cane sites, DIN concentration at the fifth percentile was 0.04 mg/l and was, on average, measured 143 days after the last application of nitrogen fertiliser. Given that this DIN concentration is within the range of the few measured observations for DIN in rainfall, we have set the lower limit in the model so that DIN concentrations in runoff cannot go below 0.04 mg/l.

The form of the three models was as follows:

For each model, runoff concentration on the date of fertiliser application was as follows:

$$ {DIN}_{\mathrm{d}1}={\mathrm{N}}_{\mathrm{i}}\times 1/k $$

1. Time and nitrogen input based model. This will now be referred to as the *time* based model.

$$ {DIN}_{rt}=\mathrm{Maximum}\ \left(0.04,{DIN}_{ry}-{DIN}_{ry}\times DL\right) $$

2. Rainfall and nitrogen input based model. This will now be referred to as the *rainfall* based model.

$$ {DIN}_{rt}=\mathrm{Maximum}\ \left(0.04,{DIN}_{ry}-\mathrm{Rain}\times RL\right) $$

3. Time, rainfall and nitrogen input based model. This will now be referred to as the *time and rainfall* based model.

$$ {DIN}_{rt}=\mathrm{Maximum}\ \left(0.04,{DIN}_{ry}-\mathrm{Maximum}\ \left(\mathrm{Rain}\times RL,{DIN}_{ry}\times DL\right)\right) $$

where

DIN_d1_: DIN concentration in runoff water on fertiliser application date (mg/l)
DIN_rt._: DIN concentration in runoff water today (mg/l)
DIN_ry_: DIN concentration in runoff water yesterday (mg/l)
DL: Daily loss proportion, fitted model coefficient
N_i_: Nitrogen input from fertiliser or plant residue (kg/ha)
Rain: Daily rainfall and irrigation (mm)
1/*k*: Fitted model coefficient to calculate DIN runoff concentration on the day of fertiliser application
RL: DIN concentration loss per mm of rainfall / irrigation, fitted model coefficient

When there were multiple fertiliser applications in a season, the new concentration was calculated as—DIN concentration from the previous day + Ni × 1/*k.*

Each model was optimised for each of the 25 seasons by minimising the mean absolute error between predicted and measured daily DIN concentrations. The parameters that were optimised in each model were as follows: *k*—divisor of the initial nitrogen fertiliser input; DL—the proportion daily loss of DIN; and RL—the loss of DIN per mm of rainfall. The optimised parameter values and the mean absolute error for each of the models are presented in Table 5.

**Table 5:** Parameter values for the three DIN models optimised for each year of measurement and the associated mean absolute error (MAE)

Year	Time			Rainfall			Time and rainfall			
	*k*	DL	MAE	*k*	RL	MAE	*k*	DL	RL	MAE
1	300	0.0052	0.04	340	0.0004	0.04	314	0.0032	0.0002	0.04
2	91	0.0349	0.08	240	0.0021	0.07	188	0.0099	0.0019	0.07
3	51	0.0266	0.14	183	0.0019	0.26	53	0.0257	0.0003	0.14
4	39	0.0213	0.06	65	0.0057	0.28	37	0.0221	0.0049	0.16
5	59	0.0142	1.92	31	0.0161	1.19	23	0.0036	0.0112	1.24
6	43	0.0062	0.95	116	0.0014	0.87	86	0.0019	0.0013	0.87
7	80	0.0399	0.28	90	0.0051	0.25	89	0.0030	0.0049	0.25
8	56	0.0340	0.13	62	0.0044	0.11	58	0.0199	0.0044	0.12
9	10	0.0400	0.30	32	0.0107	0.09	29	0.0026	0.0103	0.10
10	10	0.0315	0.49	26	0.0074	0.19	24	0.0020	0.0073	0.19
11	10	0.0367	0.04	133	0.0016	0.01	54	0.0011	0.0043	0.01
12	169	0.0123	0.07	265	0.0004	0.03	219	0.0024	0.0004	0.03
13	10	0.0338	0.12	187	0.0005	0.06	52	0.0020	0.0041	0.11
14	139	0.0129	0.04	100	0.0007	0.07	118	0.0020	0.0004	0.02
15	10	0.0312	0.19	266	0.0003	0.17	160	0.0020	0.0005	0.14
16	62	0.0459	0.21	90	0.0039	0.20	89	0.0010	0.0039	0.20
17	59	0.0518	0.51	74	0.0026	0.43	68	0.0098	0.0023	0.43
18	32	0.0670	0.35	71	0.0025	0.30	73	0.0013	0.0024	0.30
19	104	0.0212	0.38	51	0.0024	0.33	50	0.0103	0.0022	0.33
20	21	0.0576	0.52	50	0.0035	0.47	53	0.0021	0.0032	0.47
21	63	0.0211	0.27	55	0.0048	0.24	46	0.0010	0.0053	0.24
22	21	0.0304	0.03	91	0.0093	0.04	93	0.0020	0.0080	0.04
23	10	0.0355	0.02	94	0.0069	0.04	50	0.0197	0.0012	0.02
24	165	0.1000	0.18	69	0.0049	0.07	66	0.0010	0.0045	0.09
25	10	0.0225	0.14	60	0.0032	0.07	51	0.0041	0.0023	0.03
Median	51	0.0315	–	90	0.0032	–	58	0.0024	0.0032	–
SD	60	0.0302		19	0.0023		18	0.0073	0.0024	
Total	–	–	7.4	–	–	5.9	–	–		5.7

*k* divisor for N input, *DL* daily loss proportion, *RL* loss of DIN per mm rainfall (mg/l/mm), *MAE* mean absolute error between predicted and modelled DIN concentrations (mg/l), *SD* standard deviation

The *rainfall* model had a lower mean absolute error than the *time* model in 19 of the 25 year combinations. Overall the *rainfall* model had a lower total mean absolute error of 5.9 mg/l compared to the *time* model with 7.4 mg/l. The *time and rainfall* model had the lowest total absolute error of 5.7 mg/l. Although the *time and rainfall* model did not have a much lower absolute error than the *rainfall* model, the optimised parameter values for *k* and RL were less variable than the *rainfall* model. In the *time and rainfall* model, the optimised parameter *k* which determines the initial runoff DIN concentration ranged from 23 to 314. As the *k* value and coefficients DL and RL were optimised at the same time, it was possible that a broad range of parameterisations for *k*, DL and RL could result in solutions similar to the optimal solution. In general, the *k* values for granular fertilisers (years 1–4, 11–15) were larger than for fertilisers applied in a liquid form (years 7–10, 16–25). Therefore, a further optimisation using the *time and rainfall* model was undertaken based on the form of fertiliser, with three fertiliser forms considered: (a) liquid, (b) granular and (c) incorporated soybean crop.

For this stage of model development, the statistical value ‘modelling efficiency’, or Nash–Sutcliffe model efficiency coefficient (Mayer and Butler 1993) was used to evaluate model performance. Modelling efficiency is considered a better indication of the ‘effectiveness’ of model predictions, with values ranging from negative infinity to positive one. A value of positive one indicates that the model is perfect at predicting the unknown value, a value of zero indicates that the mean of the observations is as good as using the model and negative values indicate that the model was worse than using the mean of the observed values. Model efficiency values greater than 0 indicate that the model provides a better estimate than using a site measured event mean DIN concentration. The parameters *k*, RL and DL were optimised using Microsoft Excel Solver to achieve the maximum Nash–Sutcliffe efficiency for the granular and liquid nitrogen fertiliser applications (Table 6). There were 2 years when soybean crop residue was incorporated into the surface soil as a source of nitrogen for the plant sugarcane crop. Twenty-two runoff events occurred in these 2 years. In one of these years, an additional 95 kg/ha of nitrogen was added as urea fertiliser 84 days after the soybean crop was incorporated. As runoff events in this year began 195 days after application, using either of the liquid or granular fertiliser parameterisations would result in minimal contribution from the urea application to surface runoff DIN concentrations. Therefore, these 2 years have been parameterised solely based on the addition of nitrogen to the soil from the soybean crops. The soybean parameters (Table 6) result in five to 10 times lower daily loss rate when compared to the fertiliser applications.

**Table 6:** The optimised parameter values for different nitrogen fertiliser forms—granular based, liquid based and soybean using the *time* and *rainfall* model

Nitrogen input	Number of years model was based on	Minimum DIN concentration (mg/l)	*k*	DL	RL	MAE	Modelling efficiency
Granular fertiliser	9	0.11	111	0.014	0.0001	0.24	0.38
Liquid fertiliser	14	0.04	54	0.0075	0.0053	0.37	0.50
Incorporated soybean	2	0.04	50	0.0015	0.0045	0.17	0.51

*k* divisor for N input, *DL* daily loss proportion, *RL* loss of DIN per mm rainfall (mg/l/mm)

The granular fertiliser model had a *k* value of almost twice the liquid fertiliser model indicating the initial availability of DIN from liquid fertilisers is almost twice that of the fertilisers applied in a granular form. The granular based fertilisers had about twice the daily loss rate compared to the liquid fertilisers, but runoff DIN concentrations did not decrease as a result of rainfall. This was also seen in the measurements of DIN at the end of season with granular fertilisers having a higher base level of runoff DIN concentration of 0.11 mg/l compared to 0.04 mg/l for the liquid fertilisers.

### Validation of the ‘granular fertiliser’ parameterised DIN model

The granular fertiliser model was tested by using an independent field site with runoff DIN measurements, which is briefly described in the methods section. In addition to the granular fertiliser DIN model, a parameterisation based on the median parameter result in Table 5 was tested. The ‘granular’ fertiliser model performed the best out of the two models tested based on both the mean absolute error and the modelling efficiency score (Table 7). Optimising the model parameters for the validation data resulted in slightly different parameterisation, with a small improvement in both the mean absolute error and modelling efficiency.

**Table 7:** Performance of two empirical DIN models on the validation data and a parameter set optimised for the validation data is presented

Model	Minimum DIN concentration (mg/l)	*k*	DL	RL	Meanabsolute error	Modelling efficiency
Median	0.04	58	0.002	0.0032	0.33	−0.29
Granular	0.11	111	0.014	0.0001	0.24	0.24
Optimised	0.11	102	0.011	0.0001	0.24	0.29

*k* divisor for N input, *DL* daily loss proportion, *RL* loss of DIN per mm rainfall (mg/l/mm)

**Fig. 5:**
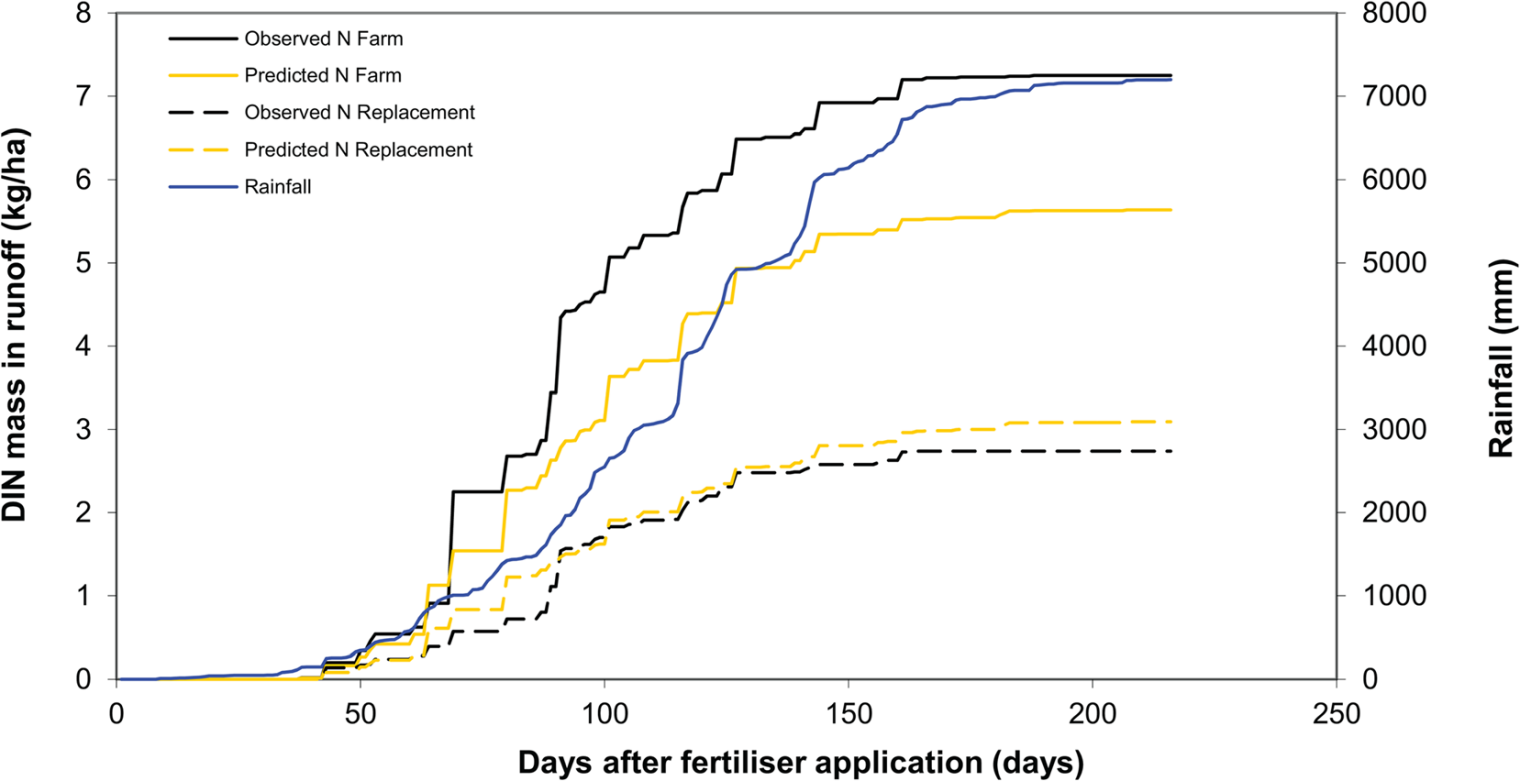
Cumulative measured and predicted (using the granular parameterisation) runoff DIN loads (kg N/ha) for the validation site N farm and N replacement treatments. Rainfall, measured and observed DIN load data have been accumulated concurrently from the date of fertiliser application which was set as day 0 for each of the three seasons

The total DIN load for the treatment with the lower nitrogen fertiliser rate (N replacement) was predicted (granular parameters) accurately; however, the load for the treatment with the higher fertiliser application rate (N farm) (Fig. 5) was under predicted. Importantly, the model represented the disproportionate contribution of DIN load from early season rainfall events (i.e. ~ 50% of the total DIN load has occurred from ~25% of the seasonal rainfall). This is an important finding from analysing the monitored DIN data and will be important for understanding the interactive effects of fertiliser application timing and seasonal rainfall conditions.

### Effect of fertiliser management practices on runoff DIN loads

**Fig. 6:**
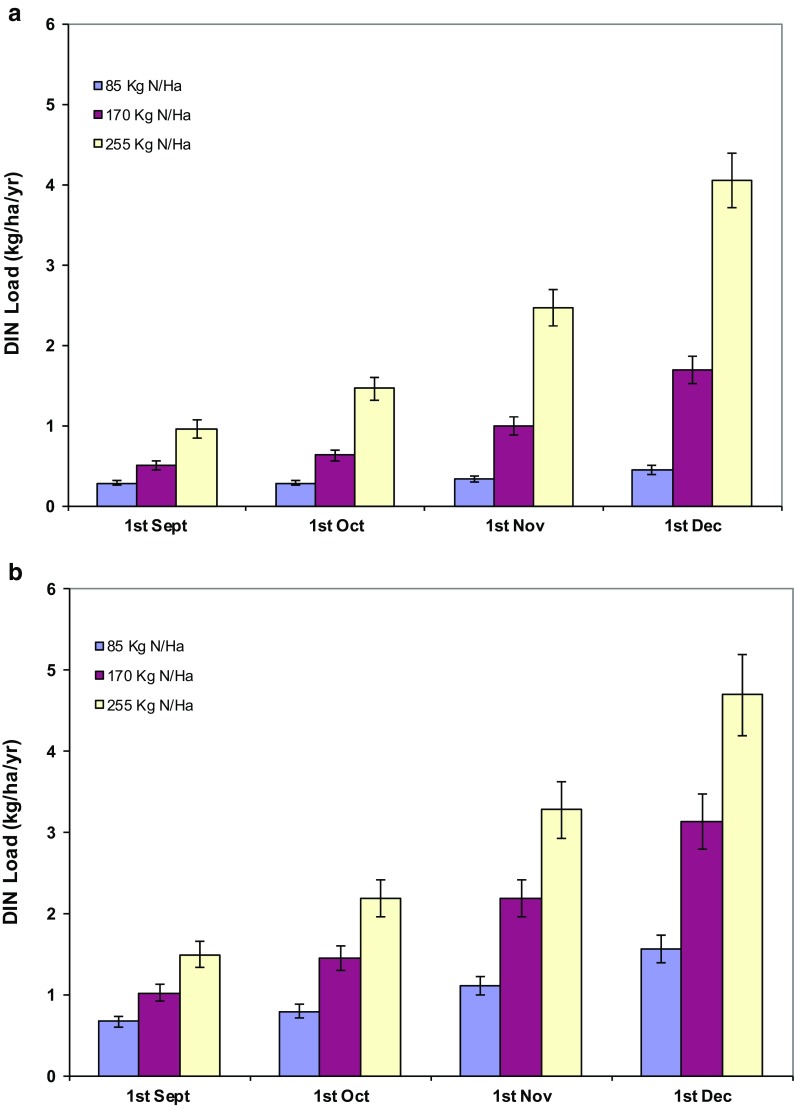
Predicted average annual DIN load and standard errors for **a** liquid and **b** granular nitrogen fertilisers applied at three rates—85, 170 and 255 kg N/ha at four different application times. Standard *error bars* are shown

The DIN model developed in this study was combined with the calibrated HowLeaky model for Victoria Plains to investigate the interaction between long-term climate conditions and DIN runoff losses. The fertiliser management practices investigated were as follows: (a) application timing (i.e. September–December), (b) nitrogen fertiliser application rate and (c) fertiliser form (granular/liquid), with results shown in Fig. 6a, b.

Application timing had only a minor effect on the rate of DIN lost in runoff for the low rate of fertiliser application of 85 kg/ha, especially for the liquid fertiliser form. For application rates close to industry standard recommended rates (i.e. 170 kg/ha), application timing did have a significant effect on DIN loads with the December applications having >3 times the runoff DIN loads compared to September applications. For the December application, the granular fertiliser (applied at 170 kg/ha) was nearly twice the rate of DIN loss when compared to the liquid fertiliser.

### Relationship between solar radiation and cane yields

**Fig. 7:**
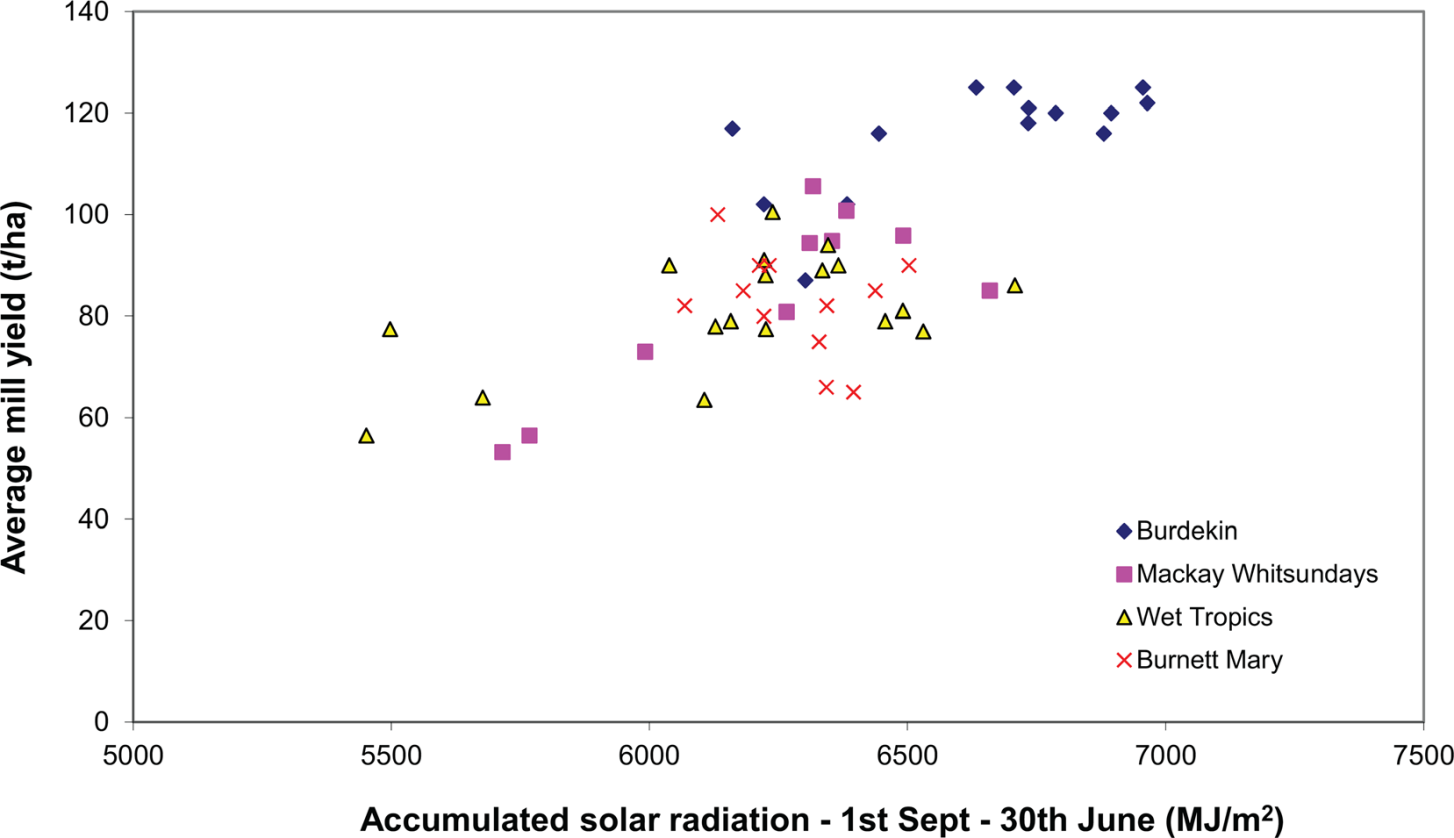
Accumulated solar radiation and average mill yield for the four major cane growing regions of Queensland

When the accumulated solar radiation is compared to the average mill yields, the derived relationship has a coefficient of determination of 0.45, *p* < 0.05 (Fig. 7). Inman-Bamber (1995) found that relatively small reductions in incident solar radiation can lead to large decreases in cane sucrose yield. The impacts of reduced solar radiation are explained as being due to two inter-relationships: firstly, a reduction in solar radiation reduces temperature that in turn slows down canopy development and its ability to intercept solar radiation, and secondly, from the direct impact of a reduction in incident solar radiation. Hence, in years that are forecast to be particularly wet with associated lower solar radiation and cane yields, reduced rates of nitrogen fertiliser application may be a management option.

### Using a seasonal climate forecast to estimate runoff DIN load risk

**Fig. 8:**
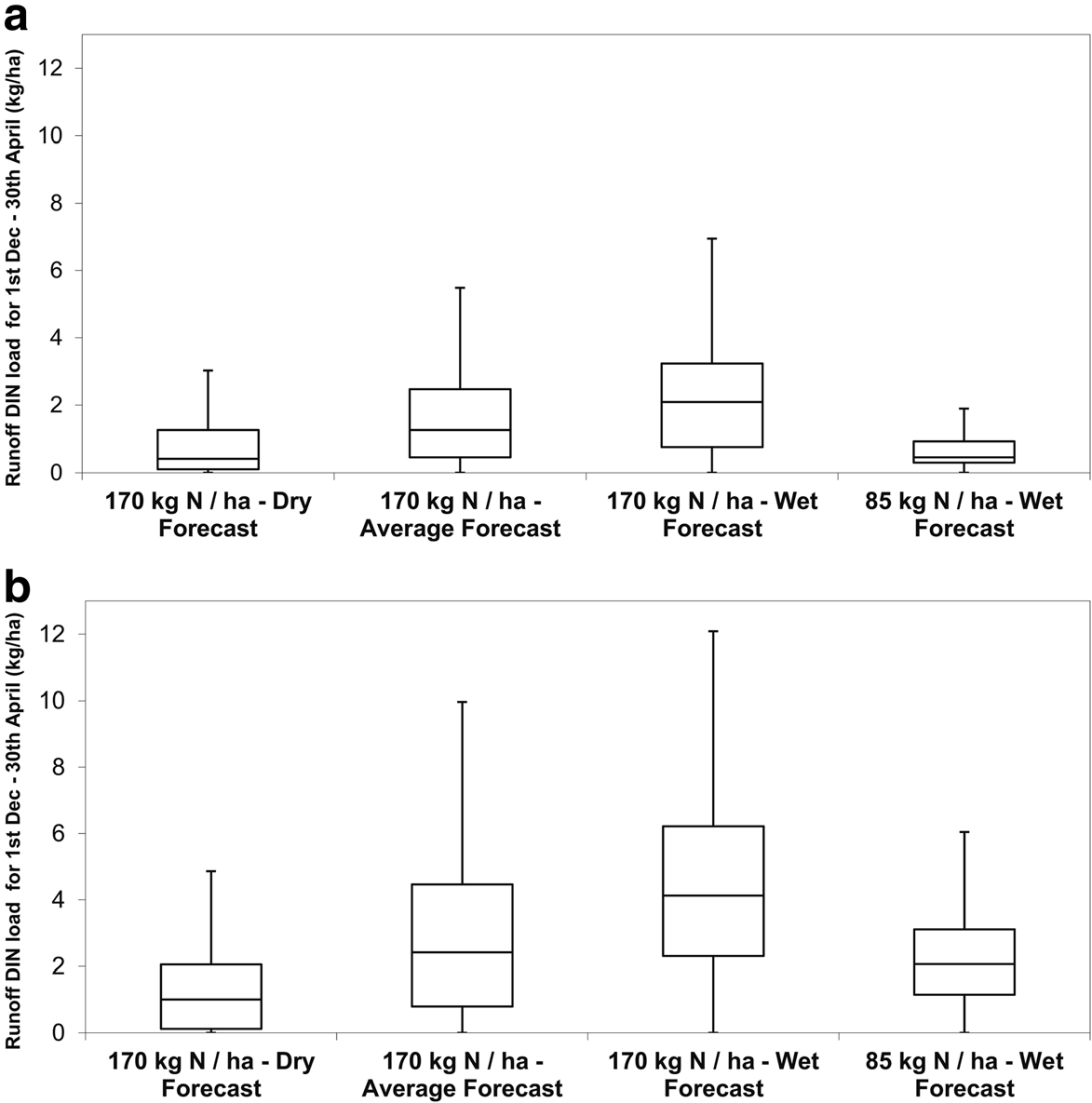
Runoff DIN load in the SPOTA-1 forecast ‘wet’, ‘average’ and ‘dry’ years when the nitrogen application rate was 170 or 85 kg/ha for **a** liquid fertiliser and **b** granular fertiliser

The median runoff DIN load in years that were forecast to be wet were 4.1 kg/ha for granular and 2.1 kg/ha for liquid fertiliser applications when applied at the 170 kg N/ha rate (Fig. 8). In contrast, the median runoff DIN loads in the combined grouping for forecast dry + average years were 1.5 kg/ha for granular and 0.8 kg/ha for liquid fertiliser applications when applied at the 170 kg/ha rate. This indicates that the median runoff DIN loads in wet years are on average 170% greater than in forecast dry + average climate years. By applying the lower rate of nitrogen fertiliser (85 kg N/ha) in this December period for wet forecast years, the median DIN loads were 2.1 kg/ha for the granular fertiliser, and 0.45 kg/ha for the liquid fertiliser. These median DIN loads were similar to those for forecast average climate years for the granular fertiliser and forecast dry climate conditions for the liquid fertiliser when applying 170 kg N/ha.

## Discussion

The daily runoff DIN concentration model developed in this study can easily be implemented in daily water balance models such as HowLeaky without the requirement to develop and parameterise a nitrogen balance model. The model has been developed from field measurements covering a diverse range of climates, soil types, nitrogen fertiliser input rates and fertiliser types. In particular, it represents the decline in runoff DIN concentrations after granular or liquid nitrogen fertiliser application, which was a key feature of the field measured dataset. This decline in runoff DIN concentrations after fertiliser application has also been observed in other field runoff studies. For example, Sharpley et al. (1982) and Barlow et al. (2007) found runoff DIN concentrations from fertilised pastures decreased greatly 1 month after fertiliser applications of 60 and ~358 kg N/ha, respectively. In contrast, the incorporated soybean crop parameterisation (Table 6) had much slower rates of decline in potential runoff DIN concentrations when compared to granular and liquid fertiliser applications. Thus, for a large soybean crop, there is a higher risk of generating high runoff DIN loads for up to 6 months after incorporation.

For the DIN model developed in this study, the rate of urea hydrolysis is thought to be an important aspect affecting the DIN model coefficient values and is believed to be the reason for the higher runoff DIN risk associated with using surface applied granular fertilisers compared to surface applied liquid fertilisers. The slower rate of hydrolysis of surface applied granular fertiliser may be due to fertiliser getting caught up in the large amounts of surface residue (e.g. 10–20 t dry matter/ha; Thorburn et al. 2001), which may limit both dissolution and metabolisation by soil bacteria. An interesting study by Prasertsak et al. (2002) found that by burying urea fertiliser in soil with sufficient moisture, hydrolysis commenced immediately and was complete after 2 days, compared to only 68% of urea being hydrolysed for surface applications. Improvements in the DIN model that explicitly represent these initial transformation processes will allow for better representation of fertiliser timing on runoff DIN and also dissolved organic nitrogen (e.g. Davis et al. 2016).

### Management options to reduce sugarcane DIN runoff loads

The simulation study results indicate the importance of fertiliser timing and seasonal rainfall conditions have on DIN runoff loads. Obviously, earlier application before the onset of the wet season reduces DIN losses; however, there are practical limits to how much the timing of fertiliser application can be altered due to the cane harvesting schedule. Sugarcane fertiliser application amounts are generally a set amount year-in and year-out with the aim of matching fertiliser N rates to crop requirements. The relationship between yield and solar radiation (Fig. 7) results presented here indicate that crop nitrogen requirements will likely vary year-to-year. Additionally, studies of isotopically labelled Nitrogen fertiliser show that of the nitrogen fertiliser applied only 1–49% is taken up by the crop (Bell 2015). These results suggest that fertiliser application rates could be reduced in high runoff risk years. Those being—when fertiliser is being applied close to the onset of the wet season and for when there is a forecast for particularly wet summer season. For the single location used in the simulation study provided here, we estimate that by reducing late season nitrogen fertiliser rates in these high risk years DIN losses from granular fertiliser were reduced by 49% and from liquid fertiliser by 78%. However, broader application and testing is recommended.

The study results suggest that the following strategies may be useful to reduce the risk of runoff DIN loads:

- Use liquid fertilisers instead of granular fertilisers
- Avoid late application of fertiliser leading into the wet season
- Irrigate (overhead) after liquid fertiliser application
- Reduce N fertiliser rates in forecast wet years

The model does not include the effects of below ground fertiliser placement compared to surface application or the impacts of banding of fertilisers on the crop row. Both of these are relatively common practices and indicate the potential for further model development. Additionally the model was developed from cane grown in predominantly rainfall fed conditions and may not be applicable for cane regions which have quite different furrow irrigation practices such as in the north Queensland dry tropics region.

## Conclusion

The daily empirical runoff DIN concentration model developed in this study can be easily incorporated into basic cropping systems models. The model allows for the effects of several key management practices (rate, time and form of fertiliser application) to be modelled. The simulation study results highlight the risk of late season fertiliser application and suggest that further investigation regarding the utility of a climate forecast to inform fertiliser management decisions be undertaken.
